# Should healthcare professionals include aspects of environmental sustainability in clinical decision-making? A systematic review of reasons

**DOI:** 10.1186/s12910-025-01230-4

**Published:** 2025-07-03

**Authors:** Sarah Gabriela Kuiter, Alina Herrmann, Marcel Mertz, Claudia Quitmann, Sabine Salloch

**Affiliations:** 1 Institute for Ethics, History and Philosophy of Medicine, Hannover Medical School, Hannover, Germany; 2 Heidelberg Institute of Global Health, University Hospital Heidelberg, Medical Faculty University Heidelberg, Heidelberg, Germany; 3 Institute of General Practice, University Hospital and Medical Faculty, University of Cologne, Cologne, Germany

**Keywords:** Climate change, Environmental sustainability, Shared decision making, Healthcare professionals, Ethics, Systematic review

## Abstract

**Background:**

Healthcare systems worldwide are large emitters of greenhouse gases and contribute to the worsening climate crisis. Attempts to reduce emissions are already being made at various levels of the healthcare system. However, the extent to which considerations of environmental sustainability should be incorporated into clinical decision-making at the individual level is unclear.

**Methods:**

We conducted a systematic review of the reasons stated for and against including aspects of environmental sustainability in the clinical decision-making of healthcare professionals. PubMed was searched as the primary data source. We screened title and abstract of all publications and performed a citation tracking of the included publications in Web of Science. All publications identified were screened for references. We performed a qualitative data analysis with a deductive-inductive approach according to Kuckartz. We used RESERVE as a reporting guideline.

**Results:**

Twenty-three publications were qualitatively analyzed in full-text. To cluster the reasons we used the four deductive categories of autonomy, beneficence, non-maleficence and justice according to the principles of biomedical ethics by Beauchamp and Childress. Additionally, the following inductive categories have emerged: autonomy transformed, non-maleficence transformed, environmental justice, professionalism, politicization, reasons addressing levels of action and an ‘other’ category. The review showed that the debate is still in its infancy and shaped by perspectives from high-income countries, while the perspective of low- and middle-income countries is lacking. For some deductively categorized reasons, a transformation towards a less individual-oriented perspective was often observed. However, it is important to recognize that the individual level is intertwined with the systemic level in the context of climate change mitigation.

**Conclusion:**

This systematic review of reasons draws attention to a possible transformation of bioethical principles, which has not yet found favor in many guidelines or codes of professional associations. It is also intended to serve as a guide for healthcare workers, policymakers and patients worldwide in developing their own stance on the issue and stresses the importance to elicit the perspectives from low- and middle-income countries.

**Supplementary Information:**

The online version contains supplementary material available at 10.1186/s12910-025-01230-4.

## Background

Environmental crises, similar to climate change, plastic pollution and biodiversity loss, are increasingly recognized as a serious threat to the health and well-being of the global population [1, 2]. Climate change leading to increasing global average temperatures, sea level rise and extreme weather events are having an immense impact on human and animal health and the integrity of ecosystems [3–5]. Many scientists now agree that maintaining global warming at below 1.5 °C relative to preindustrial levels is no longer a viable objective in face of the ongoing emissions of climate-altering greenhouse gases (GHG) [6, 7]. In order to stay within the Paris Agreement’s target of keeping global warming below 2 °C, immense and rapid efforts are needed to reduce GHG emissions across all sectors of society [7].

The healthcare sector is a significant contributor, accounting for around 4.6% of total global GHG emissions [8]. Questions of responsibility and environmental justice arise when comparing the emissions from healthcare in high-income countries with those of low- and middle-income countries. The healthcare systems of the latter two emit far fewer GHG emissions, but are particularly vulnerable to the effects of global emissions and the resulting global warming [9]. Responding to climate change will require a transformation of the healthcare sector, for instance, at the macro level of healthcare politics and the meso-level of institutional policies. The English National Health Service (NHS) set itself the goal in 2008 of continuously reducing GHG emissions, and declared in 2020 its intention to be the first healthcare system in the world to become carbon neutral by 2045 [10]. In 2019, a reduction of the carbon footprint by 26% was achieved in comparison to the 1990 baseline, thus, establishing the NHS as a pioneer in the realization of these targets [11]. Several other healthcare organizations worldwide have implemented initiatives that include commitments to climate neutrality [12]. The nongovernmental organization ‘Healthcare without Harm’ has established a supra-regional network of ‘Global Green and Healthy Hospitals,’ and is dedicated to sustainability without compromising patient care [13].

However, it is not only macro- and meso-level actions that need to be considered. Individual clinical decision-making has also been identified as a factor contributing to the emission of GHG. Impressive reductions in GHG emissions can be achieved by, for example, switching inhalers for asthma or chronic obstructive pulmonary disease from metered-dose inhalers (MDI) to dry-powder inhalers (DPI) [14]. Similarly, the consumption of N_2_O, a potent GHG used as an inhalational painkiller during labor or in the emergency department, was reduced by up to 98% in a hospital based in the United Kingdom [15]. However, such decisions cannot be made by healthcare professionals alone; the patient-provider relationship is one key element in such complex decision-making situations. Changes concerning the communication with patients might be required to promote an environmentally sustainable practice. These changes may have important ethical implications. It is unclear, for example, to what extent, if at all, it is permissible to modify the concept of patient autonomy, which traditionally implies respecting patients’ values and interests as they are, in light of the climate crisis. This could involve educating patients about sustainability as a form of patient empowerment and influencing their values, as “the immediately communicated values of the patient may not necessarily represent their true values nor best interest” [16]. Similarly, it is often unclear how to respond to a patient who declines a specific therapy due to environmental concerns. In clinical consultations, a physician may feel the need to deal with trade-offs between the patient’s preferences and optimal environmental protection, as they do not necessarily overlap. In a survey, three-quarters of US primary care providers were open to discussing climate change issues in shared decision-making, but over 60% feared it could affect their relationship with patients [17]. According to recent research, patients in Germany were open to discuss climate change and health-related content during a consultation if they were relevant to their individual health [18, 19]. This leads to the question of what ethical reasons there might be to include aspects of environmental sustainability in clinical decision-making or not.

The normative debate on (not) incorporating environmental sustainability into medical consultations is still in its infancy. To date, there have been few publications exploring the topic and discussing reasons for or against such a practice [18, 20, 21]. However, there has been no systematic overview so far. The aim of this systematic review of reason (SRoR) is to provide a full tableau of the reasons published on the ethical question whether healthcare professionals should address aspects of ecological sustainability in clinical decision-making. The review aims at providing guidance to healthcare professionals who are wondering how to deal with the climate crisis in everyday clinical decision-making. It might also inform patients as well as institutional and political policymakers who are dealing with the ecological sustainability of healthcare.

## Methods

A SRoR is an established method in bioethics research to provide an outline of the current discourse on an ethical issue [22, 23]. This SRoR provides a comprehensive overview of the reasons published in academic journals on whether healthcare professionals should address issues of environmental sustainability in clinical decision-making, including prevention, prediction, diagnosis, therapy and rehabilitation. The research question is therefore not directed at climate-sensitive health counselling (CSHC), which focuses on all implicit and explicit communication about climate change and health issues including adaptation to health impacts of climate change [24].

The article follows the RESERVE statement as a reporting guideline for SRoRs in ethics, see Supplement 5 [23].

### Search strategy and eligibility criteria

We defined eligibility criteria before conducting the database search. The research question was operationalized in four semantic clusters: healthcare professionals, environmental sustainability, shared/clinical decision-making and ethical reasons. We included only publications from academic journals written in English or German published from inception of the database PubMed in 1996 until January 8, 2024, addressing all four semantic clusters. There was no restriction on text type. The reason for these rather broad inclusion criteria was to ensure that any article dealing with this emerging topic was found. An important exclusion criterion referred to publications dealing with CSHC in general, as this concept does not necessarily relate to clinical decision-making in terms of the environmental sustainability of treatments. A full list of eligibility criteria can be found in Table 1; there were no differences between criteria for title/abstract and full-text screening.

**Table 1:** Inclusion and exclusion criteria as applied in the title and abstract and full-text screening

Eligibility Criteria		
Publication date	From inception of the database in 1996 until January 8, 2024	
Language	English or German	
	*Inclusion*:	*Exclusion*:
Publication type:	Articles published in a scientific/scholarly journal (no restriction on text type)	Books, book chapters, newspaper articles, grey literature
Content:	Publications addressing all four semantic clusters of the research question: - healthcare professionals - environmental sustainability - clinical decision-making - ethical reasons	- pediatric care or people without the ability to consent - adaptation to climate change - disaster management - Global health/Public health concepts - CSHC in general, e.g. how to cope with the climate crisis

**Box. 1:**
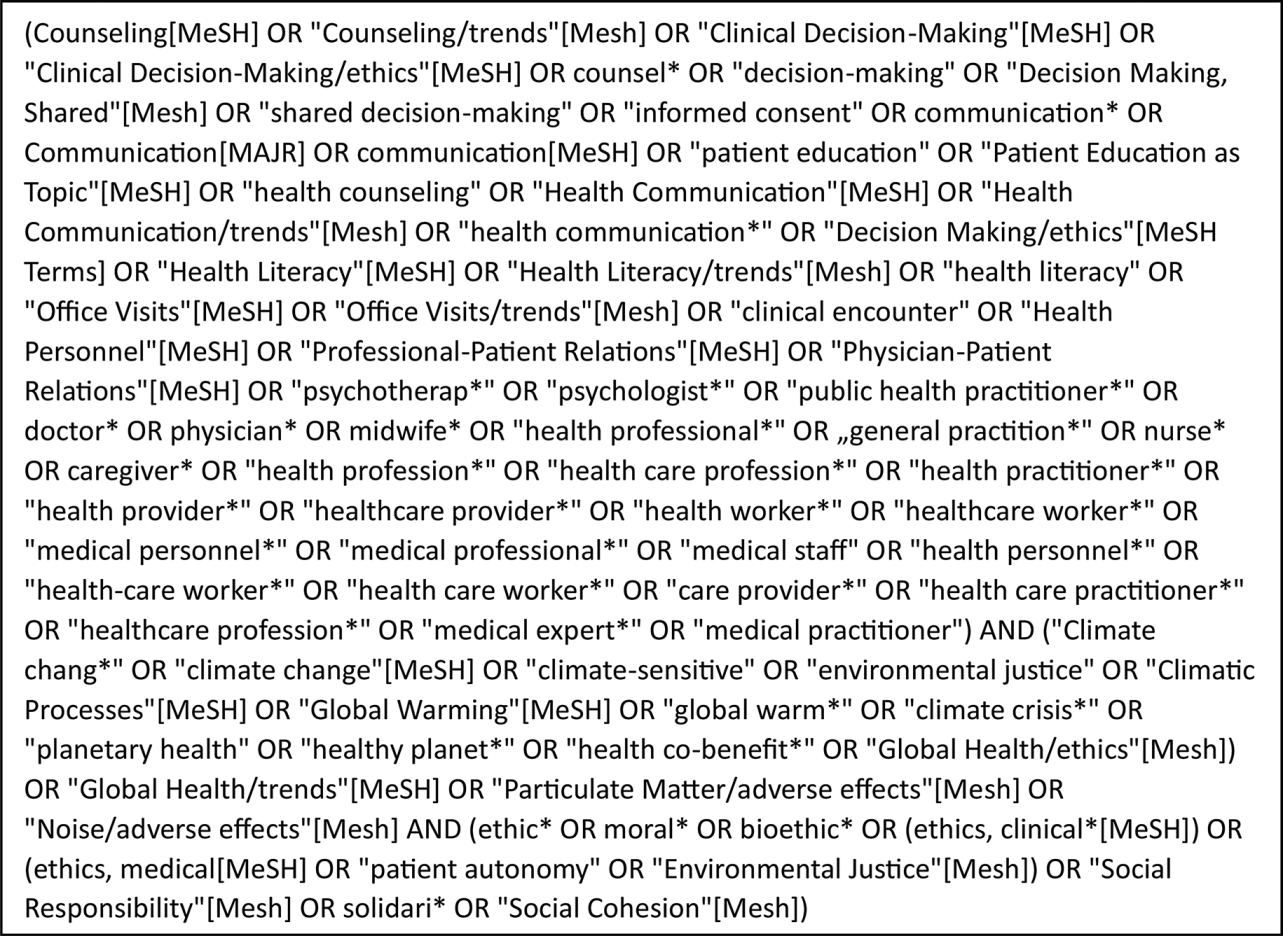
PubMed search string

In order to identify all relevant scientific publications, we initially consulted the databases PubMed, Web of Science, Google Scholar and PhilPapers. The first three databases are well established for (medical) scientific topics and very comprehensive. PhilPapers is a database for philosophical publications and was considered to include the philosophical aspects of the debate. Authors SGK and MM developed a search string for PubMed (see Box 1). The search string was applied to PubMed on January 8, 2024. We could not generate a viable search string for Web of Science and PhilPapers that would have allowed a meaningful balance between sensitivity and the specificity of the search, probably due to the novelty of the topic, the very generic nature of Web of Science or, in the case of PhilPapers, the medical-practical focus of the research question. We modified the search string for an exploratory search in Google Scholar, but the title and abstract review of the initial ten pages (out of a maximum of circa 1,000 pages, depending on the adaptation of the search string) did not produce any publications that met the eligibility criteria, aside from those previously identified. Consequently, we decided against further systematic development and execution of the search in this database. Additional publications were identified by hand searching.

### Title and abstract and full-text screening

SGK performed a title and abstract and full-text screening of the publications identified via PubMed. Due to the rather low yield of publications, the authors decided to perform a citation tracking of all publications included in the full-text screening. This was performed in Web of Science and SGK screened the publications identified based on the title/abstract and full-text, respectively. SS was consulted in unclear cases during the title and abstract screening, and agreement was reached. SGK title-screened the references of all publications that were finally included in the full-text screening. If the title addressed the research question, an abstract screening was also carried out.

### Full-text data analysis and synthesis

We extracted the following descriptive information from the publications: year of publication, first author’s name, first author’s affiliation, first author’s specialization/profession, journal name, text type (both as indicated by journal and clustered by SGK), topic addressed, language, profession addressed and form of scientific quality control (review method).

SGK extracted data from the publications in line with key principles of qualitative data analysis according to Kuckartz [25] using MAXQDA 2020 as software. Each section of a publication was generally accessible for analysis.

We used a mixed deductive-inductive approach in the full-text analysis to categorize the reasons identified in the publications; corresponding text passages were extracted. We derived four deductive categories from Beauchamp and Childress’ standard book on biomedical ethics [26], by using their interpretation of the ethical principles of respect for patient autonomy, non-maleficence, beneficence and justice. We chose this theoretical approach as the review question addresses issues of clinical ethics in the immediate provider-patient encounter. SGK inductively added further categories during data analysis, to remain open to other aspects. As the analysis progressed, narrower codes were added to complement the different main categories as accurately as possible. AH, CQ and SS advised on the development of categories and revised ambiguities.

Due to the lack of methods for quality assessment in normative literature [27], the authors did not carry out a quality appraisal. All publications included were published in scientific journals and therefore assumed to be of a certain quality based on peer or editorial review.

Patient and public involvement was not applicable to our study format.

## Results

### Overview of the sample

**Fig. 1:**
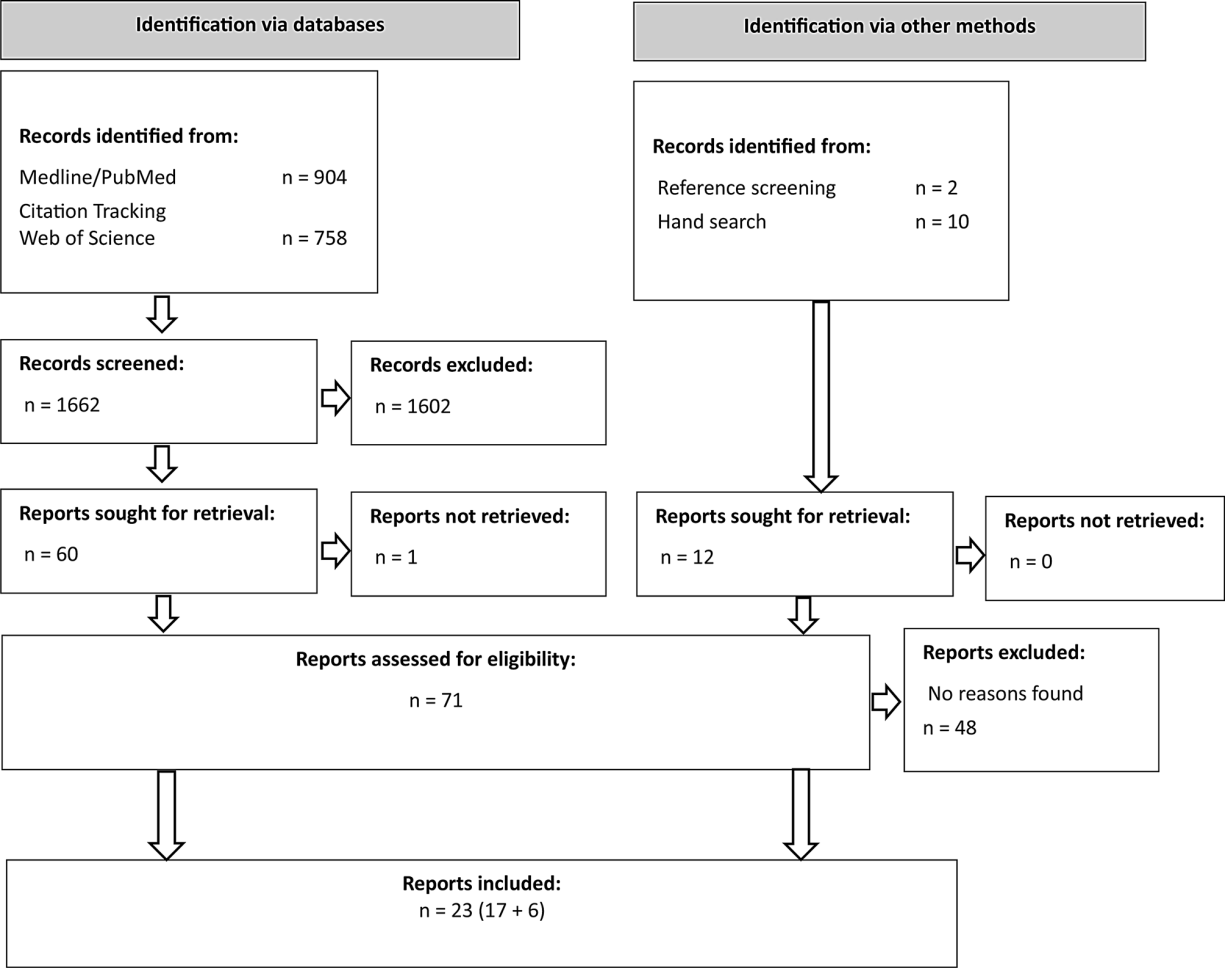
Flow diagram for study selection

The search in PubMed yielded 904 publications. Citation tracking of PubMed full texts in Web of Science identified a further 758 publications. Consequently, 1,662 publications were screened by title and abstract. After this first screening stage, 1,602 publications were excluded, one publication could not be retrieved. A total of 59 full-text publications were screened, of which 17 met the inclusion criteria. In addition, 12 publications were identified via hand search and reference screening of included publications. After screening the title and abstract, six of these publications were excluded in the full-text screening. Finally, 23 publications were included in the analysis (see Fig. 1).

An overview of the metadata of the 23 publications included can be found in Table 2, additional information can be found in Supplement 2. Notably, all first authors are affiliated with institutions in high-income countries. Table 2 shows the topics of the publications as clustered by SGK. There is a clear dominance of the debate on inhalers mentioned above, which concerns the use of more climate-friendly DPIs instead of MDIs for chronic lung diseases. Furthermore, there is a debate on professionalism concerned with the attitudes of healthcare professionals towards addressing ecological sustainability, particularly regarding the arguments of professional associations and guidelines on the subject. The reproductive medicine debate focuses on the arguments of antinatalism, as a topic of contemporary ethics that is gaining importance in light of the climate crisis. Its main argument is against procreation, as an increase in the global population results in elevated GHG emissions, thereby intensifying the climate crisis [28].

**Table 2:** Characteristics of publications included

Characteristics	Subgroups	Number of articles in the sub-groups (*n*)
Year	2024 (Search string applied on January 8, 2024)	1
	2023	14
	2022	3
	2021	1
	2020	1
	2017	1
	2009	1
	2008	1
Article type	Argumentative text	9
	Commentary	7
	Case presentation	2
	Quantitative data analysis	2
	Informative text	2
	Editorial	1
Country*	United States of America	8
	United Kingdom	6
	Switzerland	1
	Norway	1
	Mexico	1
	Sweden	1
	Belgium	1
	Netherlands	1
Journal	Journal of Medical Ethics	10
	Medicine, Health Care and Philosophy	3
	Journal of the American Board of Family Medicine	2
	AMA Journal of Ethics	2
	British Medical Journal	1
	British Journal of General Practice	1
	Die Dermatologie	1
	Hebamme	1
	International Journal of Environmental Research and Public Health	1
	The Lancet	1
Review mode	Peer Review	10
	Editorial review	10
	No peer review	3
Group of healthcare professionals addressed**	Physicians	18 Physicians in general (14) General practitioners (4) Gynecologists (3) Dermatologists (1)
	Bioethicists	12
	Midwives	1
	Global Health Professionals	1
Topics**	General	13
	Inhaler	7
	Professionalism	3
	Reproductive medicine	3
	Gynecology	2
	Dermatology	1

*As one author was affiliated with two countries, the total number is higher than the number of studies included

**Several studies addressed more than one group, respective topic

A total of 67 ethical reasons for, against or ambivalent regarding the research question were identified. Thirty reasons were identified in favor of integrating aspects of environmental sustainability into clinical decision-making. Conversely, 27 reasons were identified against implementing such measures, while 10 reasons were assessed as ambivalent as the authors’ tendency of using these reasons could not be clearly determined. A comprehensive table of reasons (Supplement material 3) and the code tree (Supplement material 4) can be found in the supplementary material.

**Fig. 2:**
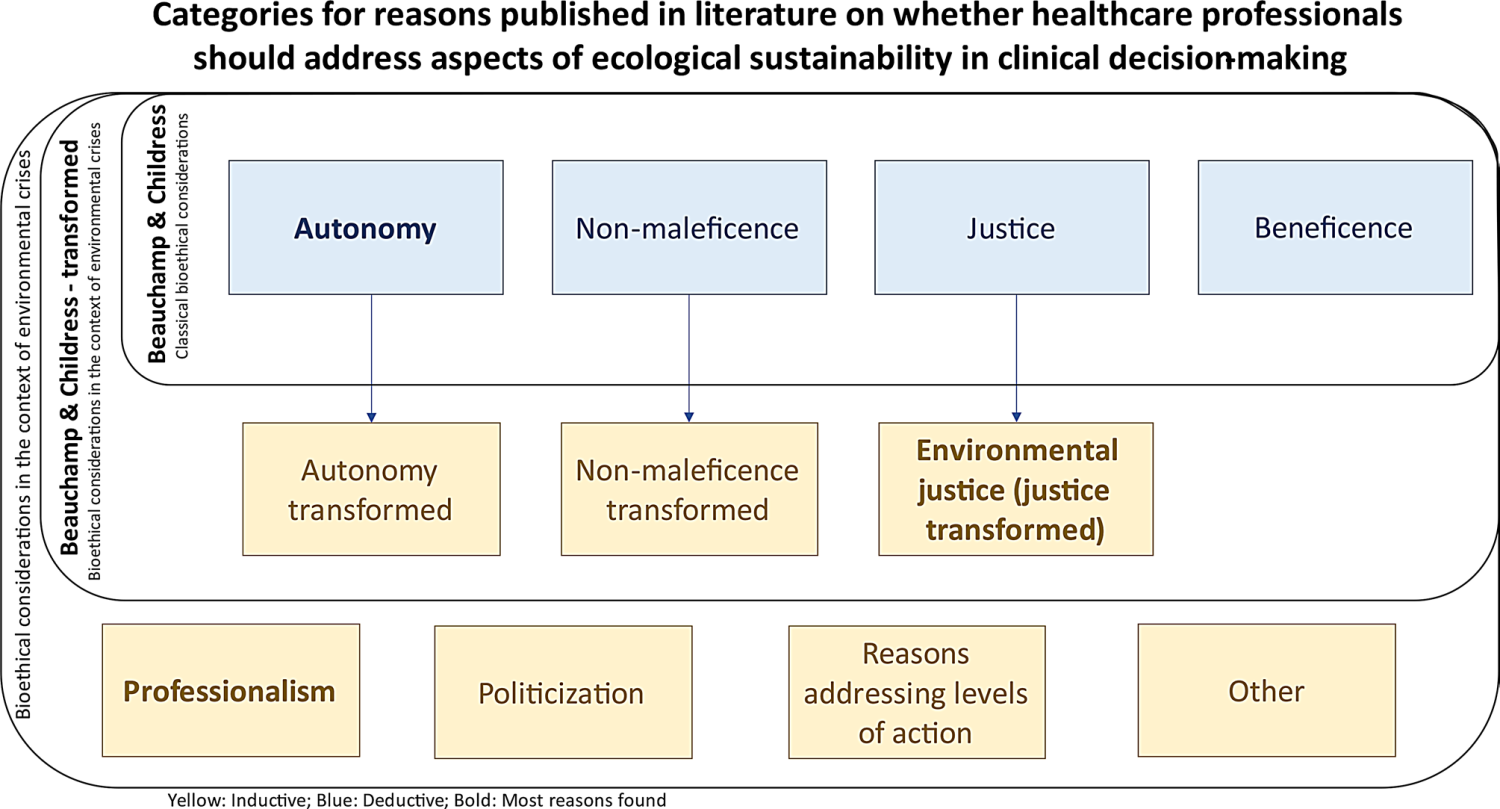
Illustration of deductive and inductive categories

### Reasons related to respect for patient autonomy

We found most reasons in the deductive category of respect for patient autonomy (see Fig. 2; Table 3), with most of those reasons being against implementing aspects of environmental sustainability in clinical decision-making. These reasons focused on the classic individualistic definition of patient autonomy, centered on one single patient, as advocated, for example, by Beauchamp and Childress. Concerns were expressed regarding the potential loss of trust on the part of patients in the physician providing care when an inhaler is switched without the patient’s consent, or an offered switch is turned down by the patient, but this wish is not respected by the physician [29]. One ambivalent reason stated that “(…) [W]hat is morally right or wrong for patients to choose is normally not seen as relevant for the issue of whether or not their autonomy should be respected” [30].

The category of shared decision-making as a subcategory of respect for patient autonomy yielded a considerable number of ambivalent reasons and reasons against. The reasons against focused on not “impos[ing] environmental protection values on [a patient’s] decision-making” [31] from the physician’s side, while the ambivalent reasons looked at the setting in which those conversations would occur (e.g., focusing on sensitive and patient-centered communication) [29] or on the necessity of decisions being made collaboratively [32, 33] – instead of paternalistically. However, the positive reasons regarding patient autonomy were found multiple times in different text segments of different publications, highlighting the fact that “a healthcare provider might be withholding information if she did not provide the patient with environmental data” [34] and, thus, potentially leading to a breach of respect for patient autonomy.

Furthermore, two text segments indicated a certain transformation of the classical definition provided by Beauchamp and Childress. One author argues that “when self-interest and inadequate resources harm others, autonomy loses integrity,” [35] which broadens the scope of patient autonomy and provides a reason for the implementation of environmental sustainability in clinical decision-making. Respect for autonomy is, therefore, not understood as an absolute means, as patients’ decisions must meet certain moral criteria in order to be considered to have integrity. This creates a tension between the principles of autonomy and justice.

### Reasons related to justice

The concern was expressed that addressing climate issues in the consultation could perpetuate injustices, as many people in the consultation belong to vulnerable groups already, especially in the debate around reproductive medicine and coerced sterilization [31]. Indeed, there are also some scholars now arguing that in light of the climate crisis, there is a duty to have fewer children, if any at all, referring to the concept of antinatalism [36–38]. Taking these reasons into account, it seems reasonable to conclude that there is a potential risk that those who have been victims of injustice in the past may be forced to forego certain medical treatments that are harmful to the climate. Again, the tension between autonomy and justice becomes apparent when a patient’s own decision-making power is potentially limited by considerations of GHG emissions of healthcare interventions.

By contrast, it was argued in a transformed understanding of justice (see Fig. 2; Table 3) that addressing environmental sustainability would align with the global public health responsibility and, thus, set the focus on the “global ethical priority” to reduce GHG emissions [34]. This would transcend the individual-oriented notion of justice, as defined by Beauchamp and Childress, and instead focus on a global idea of justice, and on public health rather than individual health. One might conclude that this could be at the expense of individual patient autonomy and instead benefit third parties, for example future generations or distant communities.

### Reasons related to non-maleficence

In terms of non-maleficence, Wiesing argues that “(…) if the best intervention is not chosen for environmental reasons and the patient is not treated optimally and worse off, then there is a serious conflict” [39]. This would mean causing or risking harm by not treating patients optimally and violating the principle of non-maleficence.

However, a broadening of the focus was also found here, where some authors pointed out that future generations also have a right to not be harmed [40]. Parker, for example, introduces the duty to “minimise expected harm,” [14] which refers to potential environmental harm resulting from healthcare interventions that negatively affect third parties (e.g. future generations), and would be accompanied by an advocacy for addressing sustainability issues with patients.

### Reasons related to beneficence

Beneficence was a rather small category, with only two, albeit positive, reasons found. It was stated in a case presentation on the debate on reproductive medicine that taking time as a doctor for a patient to “(…) consider […] the potential environmental impact of a subsequent pregnancy and whether it is acceptable to bring a new child into the world at this time is in accordance with the principle of beneficence” [31].

### Reasons related to professionalism

The most comprehensive inductive category is that of healthcare professionalism (see Fig. 2; Table 3). While reasons regarding the understanding of physicians’ roles were frequently mentioned, other health professions were only addressed rarely. Reasons against the implementation of sustainability aspects included concerns that there would be an undue burden of responsibilities on health professionals and that the physician-patient relationship is ill-suited for the resolution of complex ethical issues that presumably cannot be resolved only at the individual level [39]. By contrast, arguments in favor of addressing climate protection include the assertion that this is simply part of the doctor’s professional remit, even now [41]. With a change in physicians’ practical identity, as has already happened in the past regarding patient autonomy as a principle, “(…) climate protection [can be] perceived (…) as part of the physician’s ‘job’ [and] raising such issues in the clinical encounter might come up more naturally and be less irritating to patients” [41].

Some reasons identified refer to the codes of medical associations. Some authors noted that a few physicians’ codes already emphasize the practice of medicine in an environmentally sustainable manner, such as the World Medical Association’s International Code of Medical Ethics [42] in its last revision of 2022 [41]. Conversely, other authors have noted that other codes of conduct explicitly prioritize the individual patient, as ten Have points out, citing the Declaration of Geneva in its 2006 version and UNESCO’s Universal Declaration on Bioethics and Human Rights, also in its 2006 version [43]. The most recent update of the World Medical Association’s Declaration of Geneva [44] in 2017 also states that “the health and well-being of my patient will be my first consideration” [44], with a similar wording to the two above.

### Reasons related to politicization

It has been noted in the debate on the politicization of medical consultations, that politicization in a physician’s office is not an uncommon phenomenon, with physicians discussing “abortion care, transgender care, sexual and reproductive health, lifestyle counselling and end-of-life counselling” [45]. Cohen et al. point out that physicians are experienced and equipped to deal with politicized issues such as the above; climate change would simply add to this list [45]. Furthermore, the discourse within the medical consultation can serve to inform the patient about the issue under discussion, which may subsequently “open opportunities for patients to consider other ways in which they can protect the environment.” [46].

General practitioners were “(…) reluctant to discuss reproductive health with patients (to limit unwanted pregnancies and population growth)” [47] only in one qualitative study with such doctors on communicating about the climate crisis with their patients. Here, the topic of reproduction was perceived as too politicized.

### Reasons related to appropriate levels of action

Looking at appropriate levels of action that could be targeted for measures in climate protection, it was pointed out that addressing the meta level of healthcare in terms of a structural or institutional approach alone will not solve all the problems, as it cannot be “divorced from the interactions that doctors have with their patients” [48].

However, the claim still stands that healthcare providers “should focus on advocating for system-level changes in health care financing, organization, and delivery whilst using discretion when bringing up environmental concerns with their patients” [49]. Furthermore, Herlitz et al. argue that focusing solely on the individual and the micro level would “weaken […] the attractiveness of [the] move towards ‘green’ bioethics” [30].

### Other reasons

Another reason against the inclusion of environmental sustainability aspects in the consultation is worth mentioning, but was only discussed once in the literature included: the difficulty of quantifying emissions, not only from the technical point of view of implementing the correct method of calculation [50, 51], but also in terms of the ethical implications, as this endeavor can be “value laden and morally complex to implement in practice” [52].

**Table 3:** Table of reasons

Main category	Subcategory (if available)	Reason	Publication(s)
Respect for patient autonomy		Origin of respect for patient autonomy (+)	[45]
		Morals are not relevant for patient autonomy (+/-)	[30]
		Reproductive medicine debate: Serious strain on patient autonomy (-)	[54]
		Overriding patient’s refusal is threat to doctor’s trust (-)	[29]
	Informed consent	Educating patients is part of “going green” (+)	[55]
		Physicians would withhold information (+)	[31], [45], [34] (2x)
		Patients should know about climate impact of healthcare measures (+)	[34]
		No need for specific information if both options are standard (-)	[55]
		Physicians should act in patients’ interest (-)	[39]
	Shared decision-making	Eliciting patient’s wishes/values to form a decision (+)	[31] (2x), [45], [40], [29]
		Patients seem to be willing to hear climate impact of healthcare measures (+)	[45]
		Unclear, what practicing sustainable healthcare really means (+/-)	[54]
		Only adequate when patient expresses interest (+/-)	[49]
		It is important how the conversation occurs (+/-)	[29]
		Sometimes, patients should not have a choice (+/-)	[49]
		Decisions should be made collaboratively (+/-)	[32], [33]
		Physicians should not impose their values on a patient (-)	[31]
		Autonomy reigns supreme if a patient is steadfast (-)	[14]
		Danger of damage to the patient-provider relationship (-)	[49]
		Physicians feel constrained (-)	[43]
		GPs haven’t been approached by patients (-)	[17]
Respect for patient autonomy – transformed		There is no vacuum around autonomy (+)	[52]
		Autonomy only as long as no other person is harmed (+)	[35]
Non-Maleficence		Avoidance of overdiagnosis or over-treatment (+)	[34]
		Maintain trust/protect patient’s health to do no harm (-)	[29]
		Doing harm in not treating patients optimally (-)	[39]
Non-maleficence – transformed		Connecting healthcare delivery with the necessity to reduce GHG emissions (+)	[40], [43], [34]
		Duty to avoid expected harm (+)	[14], [29] (2x)
Beneficence		Co-benefits of “green prescribing” (+)	[29]
		Help patients to find out what they want (+)	[31]
Justice		Higher cost to reduce GHG emissions but no direct difference for patients (+/-)	[29]
		Possibility of perpetuating injustice due to counselling (-)	[31]
Environmental justice (justice transformed)		Duty to accept higher costs due to wider health benefits for low- or middle-income countries (+)	[29]
		Conservation of good living standards for everyone (+)	[61]
		Acknowledging local public health responsibility/ global health responsibility (+)	[52], [35], [45], [34], [41]
		Health consequences address all affected parties (+)	[52]
		Patients save GHG emissions for doctors to fly around the world to attend conferences (-)	[38]
	Polluter pays	Whoever causes harm needs to fix the problem (+)	[29]
		Who exactly is the polluter? Difficult to find out (-)	[29]
		Subsistence emissions are exempt (-)	[29]
Politicization		Discussion makes patients aware of the climate crisis (+)	[45] (2x)
		Politicization is no unusual phenomenon for clinicians (+)	[45]
		Reproductive issues are too sensitive and controversial (-)	[47]
Reasons addressing levels of action		Only addressing the meta level does not solve all the problems (+)	[48]
		Pharmaceutical/chemical products as biggest contributor to climate crisis within healthcare sector (+)	[43]
		The focus on the individual weakens argumentation (-)	[30]
		It is not the physicians’ task to decide (but society’s) (-)	[39]
		Providers should focus on systems-level approaches (-)	[49]
Professionalism	Healthcare professionals’ role in general	“Reasonable people” care about the climate crisis, healthcare professionals are included in that group (+)	[45]
		Ethical imperative of planetary health principles (+)	[61]
	Physicians’ role	Knowing and teaching climate protection is medical expertise (+)	[52], [46] (2x), [32] (3x), [41] (3x)
		Trust is built while disclosing climate-related information (+)	[45], [46]
		Meddled responsibilities (-)	[39]
		Patient’s wish comes first (-)	[39]
		Physicians’ obligation to confidentiality (-)	[39]
		Trust might be lost (-)	[39]
		Important ethical decisions must be made outside of the physician-patient relationship (-)	[39]
	Professions’ codes / initiatives	Physicians’ codes already recommend practicing in environmentally conscious ways (+)	[54], [41]
		Changing codes might be seen as normatively binding (+)	[41]
		Physicians hold responsibility over a single patient (-)	[43], [39]
Other		Epistemic openness (+)	[61]
		Complexity of cause-effect relationship of climate crisis (+/-)	[32]
		Dependence on topic of discussion in consultation (+/-)	[45]
		Ethical conflicts between individuals and public (+/-)	[32]
		Technical difficulties quantifying emissions (-)	[52]

List of abbreviations:

(+) = positive reason, for implementing aspects of environmental sustainability in clinical decision-making

(-) = negative reason, against implementing aspects of environmental sustainability in clinical decision-making

(+/-) = ambivalent reason regarding implementing aspects of environmental sustainability in clinical decision-making

The frequency of the reason identified in the publication is indicated after publication number (for example: Publication [14] (3x)); if no number of reasons is indicated, the reason was identified once in the named publication

See Supplement material 1 for a list of articles included in the review in alphabetical order.

## Discussion

In this SRoR, we investigated healthcare professionals’ reasons for including or not including environmental sustainability considerations in clinical decision-making. We found about as many reasons in favor as against, with some further reasons being classified as *ambivalent*. According to the results, for some reasons, there might be a notable shift in the traditional bioethical principles of respect for patient autonomy, non-maleficence and justice (see Fig. 2). Those reasons were mostly in favor of including environmental sustainability considerations. The other reasons found were mostly clustered around the topics of professionalism, politicization and reasons related to different levels of climate action. There, the reasons were more balanced. It also emerged that the debate is still in its infancy and, therefore, characterized by circumscribed discourses that are very much in the foreground, such as the inhaler debate [29, 30, 33, 46, 49].

A transformation of the classic Beauchamp and Childress principles was observed, except for beneficence, although the extent of the change varied in terms of the number and complexity of reasons. The overall change can be described as a shift away from patient autonomy as it is traditionally understood toward a focus on the individual in relation to their environment, with implications for all four principles. Christina Richie’s book *Principles of Green Bioethics* [53] reflects this shift as well. Her four principles culminate in the practice of *green informed consent* which “offers environmental information about the diagnosis and possible medical interventions” [34] of a clinically necessary procedure, recognizing the clinical and personal values of the patient, in order to find the medical option that fits best to the patient.

Both the transformed principles as well as Richie’s green principles reveal a notable lack of focus on the individual. If we refer to clinical decision-making, however, the individual dimension cannot be ignored in its interaction with powerful factors at the institutional and system levels of healthcare. After all, the system level consists of individual professionals making decisions and taking action regarding the climate crisis. This is reflected in the questions that have already been raised in the scientific discourse: How should healthcare providers deal with patients who refuse to switch from an MDI to a DPI [29]? Is the use of assisted reproductive technologies fully justified in the face of the climate crisis, especially when they are subsidized by the state [54]? Can the use of disposable products be fully justified [55]? Furthermore, the scenario first mentioned highlights the tension between the principles of autonomy and justice: a patient who opts not to switch from a MDI to a DPI may contribute to emissions that exacerbate climate change, even though they cannot be held solely accountable and their individual emissions are relatively small. Framing emission reductions not only as climate change mitigation but also as a contribution to disease prevention may prove beneficial in this context. This approach would be consistent with the principle of beneficence [56].

Regarding the systems level, discourses on professionalism and health policy were discussed in the publications. The most recent versions of the statutes and guidelines adopted by professional associations, such as those published by the General Medical Council [57] in 2024, increasingly address environmental sustainability aspects. Nevertheless, critics maintain that these amendments do not go far enough [58]. Indeed, it can be argued that the individual level, the provider-patient relationship, is often omitted or only implicitly included, and that the guidelines tend to focus on the macro level with vague formulations, for example, when it is mentioned that healthcare professionals “should consider supporting initiatives to reduce the environmental impact of healthcare” [57]. The World Health Organization has published several documents related to sustainability and health, but recommendations for a more climate-sensitive healthcare system mostly remain at the system level as well [59, 60].

The Planetary Health Pledge by Wabnitz et al. [61] represents an exception, with the difference that it is initiated by individuals rather than a professional association. Nevertheless, this pledge addresses the individual level and asks healthcare professionals to adopt an integrative approach to the issue. In Germany, a specific guideline explicitly encourages climate-conscious prescription of inhalers [62]. Additionally, a guideline entitled “Climate-sensitive health advice for GP practices” has been formally documented and is scheduled for completion by the end of 2025 [63]. Apart from the two aforementioned guidelines, however, there is currently a lack of guidelines in German-speaking countries that explicitly address climate-sensitive health advice. In the United Kingdom, a set of guidelines has been developed for ‘greener surgery’. These guidelines include a multi-stage checklist that is intended to focus on sustainability at different stages of a surgical procedure in the operating theater [64]. Guidelines like these are a first step in raising awareness about climate-conscious health measures. However, there is currently a lack of guidelines that focus specifically on how to communicate these options to patients. In addition to the development of further guidelines in this area, the implementation of compulsory modules on environmental sustainability in healthcare at the university level would ensure in-depth knowledge transfer. Some universities already offer non-compulsory courses (mostly on the topic of planetary health) [65, 66], but in order to transfer knowledge across the board, such modules would need to be included in the respective national catalogues of learning objectives for human medicine.

### Implications for research and practice

An important aim of this study is to provide healthcare professionals with guidance on how to address the climate crisis in everyday clinical decision-making by presenting a comprehensive overview of the reasons published in scientific articles. The following section contains synthesized, concrete guidance as derived from the reasons identified in the included publications.

Since the first authors of all publications are affiliated with institutions in high-income countries, the debate about whether to address environmental sustainability in clinical consultations may currently be very much framed by a “Western” perspective. It would be important to integrate perspectives from low- and middle-income countries in future research. Although healthcare systems in those countries currently emit significantly less GHG emissions than those in high-income countries [8], it is important to strengthen the capacity of their healthcare systems in a climate-friendly way. Therefore, the topic of addressing environmental sustainability in clinical consultations is important for healthcare systems worldwide. Recent articles demonstrate an interest in this topic [67]; however, further research is necessary to examine the ethical challenges associated with these issues, particularly for vulnerable and indigenous groups in regions with limited resources. Conversation and communication with patients regarding the environmental implications of prevention, diagnosis, and therapy are also necessary. Accurate data on emissions from medical treatments is essential to effectively address this matter in consultations. Therefore, future empirical research should aim to fill the existing evidence gaps on the environmental impacts of medical care, particularly GHG emissions. Ethical analyses should explore the potential transformation of classical bioethical principles, particularly autonomy and justice, and elucidate the inherent tensions between them. Furthermore, the role of healthcare professionals in the context of the climate crisis should be explained with particular attention paid to the tensions between individual and system-level actions. Given the serious consequences of the climate crisis and the specific challenges faced by healthcare providers [48], it would be beneficial to develop a compelling set of guidelines. This should be done by a scientifically legitimate and broadly representative body so that the guidelines might be perceived as normatively binding and capable of bringing about genuine change within the medical profession, thereby, supporting healthcare professionals in their clinical routines.

Practice implications for healthcare professionals include being open to patients’ preferences within the shared decision-making process. This entails taking sufficient time for consultations and being mindful of the overall setting. The setting should be calm, safe and supportive. Under no circumstances should healthcare professionals impose their own values and preferences on patients. Instead, they should carefully consider the patient’s situation and assess whether considerations of environmental sustainability might be pertinent. This may depend on factors such as diagnosis, treatment indication and patient preferences. It is, therefore, beneficial for healthcare providers to have established a strong relationship with the patient in advance. Healthcare providers may use established tools such as the Choosing Wisely questions to encourage patients to participate more actively in the shared decision-making process [68]. Regarding the powerful structures at the levels of healthcare systems and institutions, healthcare professionals should be encouraged to develop an ethically sound position on the inclusion of environmental sustainability. They should also be motivated to participate as professional actors in political and social discourse, especially within professional associations, to advance the development of new and concrete guidelines on this topic.

### Limitations

One limitation of this work is the difficulty in identifying all pertinent publications due to inadequate indexing in the medical databases, which is probably due to the novelty of the topic and the fact that no scientific consensus on terminology has yet been found. Furthermore, including only English and German publications imposed language restrictions. In addition, the perspectives of non-physician professions could only be included to a limited extent. This may be attributable to the semantic cluster “clinical decision-making” in the research question which fits primarily to physicians. It is important to note that this SRoR does not assess the quality of the arguments presented, but rather provides a comprehensive overview of the current scientific discourse. Consequently, arguments and reasons can be utilized as a foundation for further critical discussion and research.

## Conclusions

This SRoR is the first, to the best of our knowledge, to investigate the reasons why healthcare professionals should address or refrain from addressing environmental sustainability considerations in clinical decision-making – an area with significant implications for the promotion of planetary health. Utilizing the four classic bioethical principles outlined by Beauchamp and Childress, we categorized and coded the reasons identified in the literature and further developed categories inductively during data analysis. The literature analysis revealed that the decision to address such considerations depends on the patient, diagnosis and circumstances, rather than providing a straightforward answer to the research question. A consolidated patient-provider relationship and a well-established position on environmental sustainability are beneficial for both healthcare providers and patients. Nevertheless, it is crucial to acknowledge that responsibility cannot be entirely delegated to the individual level. In the future, professional organizations should develop guidelines that offer specific recommendations for addressing such issues at the individual level. Universities should offer mandatory courses on the environmental impact of healthcare to familiarize the next generation of healthcare professionals with the issue. Collaborative efforts across all levels of action, including the global level, are imperative to effectively combat the climate crisis and advocate for planetary health.

## Electronic supplementary material

Below is the link to the electronic supplementary material.

Supplementary Material 1

Supplementary Material 2

Supplementary Material 3

Supplementary Material 4

Supplementary Material 5

## Data Availability

Data collected for analysis included all coded text passages that represented reasons concerning the research question. These data will be made available on reasonable request to sarah.kuiter@stud.mh-hannover.de.

## Abbreviations

GHG: Greenhouse gases
NHS: National Health Service (of England)
MDI: Metered-dose inhalers
DPI: Dry-powder inhalers
US: United States (of America)
SRoR: Systematic Review of Reason
CSHC: Climate-sensitive healthcare counselling
RESERVE: Reporting of systematic reviews in ethics
UNESCO: United Nations Educational, Scientific and Cultural Organization
