# Supplementary material for: Should healthcare professionals include aspects of environmental sustainability in clinical decision-making? A systematic review of reasons
Source: BMC Med Ethics. 2025 Jul 3;26:78. doi: 10.1186/s12910-025-01230-4 (PMC12226885; doi:10.1186/s12910-025-01230-4)
Supplement: Supplementary file 1 — Supplementary Material 1 [file 12910_2025_1230_MOESM2_ESM.pdf]

Supplement 1: Articles included in the review

1 André H, Gonzalez Holguera J, Depoux A, et al. Talking about Climate Change and Environmental Degradation with Patients in Primary Care: A Cross-Sectional Survey on Knowledge, Potential Domains of Action and Points of View of General Practitioners. *Int J Environ Res Public Health* 2022; **19.** https://doi.org/10.3390/ijerph19084901.

2 Bhopal A, Baeroe K. Dual duties to patient and planet: time to revisit the ethical foundations of healthcare? *J Med Ethics* 2023; **49:** 102–03. https://doi.org/10.1136/jme-2022-108847.

3 Brown BP, Chor J. What Are Risks and Benefits of Not Incorporating Information about Population Growth and Its Impact on Climate Change into Reproductive Care? *AMA J Ethics* 2017; **19:** 1157–63. https://doi.org/10.1001/journalofethics.2017.19.12.ecas1-1712.

4 Chambers JC. Doctors and climate change: Impact of medical ethics. *BMJ* 2008; **336:** 291–92. https://doi.org/10.1136/bmj.39479.533125.3A.

5 Cohen ES, Kringos DS, Hehenkamp WJK, Richie C. Harmonising green informed consent with autonomous clinical decision-making: a reply to Resnik and Pugh. *J Med Ethics* 2024. https://doi.org/10.1136/jme-2024-109863.

6 Coverdale J. Green inhaler prescribing and the ethical obligations of physicians. *J Med Ethics* 2023; **49:** 99. https://doi.org/10.1136/jme-2022-108848.

7 Dresen F, Bechert S, Bolkenius D, Snyder-Ramos S, Koch S. Lachgas-Analgesie unter dem Aspekt: Nachhaltigkeit im Kreißsaal. *Hebamme* 2023; **36:** 53–61. https://doi.org/10.1055/a-2160-4933.

8 Have H ten, Gordijn B. Green bioethics. *Med Health Care Philos* 2023; **26:** 497–98. https://doi.org/10.1007/s11019-023-10182-8.

9 Herlitz A, Malmqvist E, Munthe C. "Green" Bioethics Widens the Scope of Eligible Values and Overrides Patient Demand: Comment on Parker. Unpublished, 2022.

10 Heuer R, Nast A. Sustainable prescription and implementation practices in clinical practice guidelines. *Dermatologie (Heidelb)* 2023; **74:** 34–40. https://doi.org/10.1007/s00105-022-05083-5.

11 Holman HT, Bouthillier MJ, Müller F. Thinking "Green" When Treating "Pink Puffers" and "Blue Bloaters"-Reducing Carbon Footprint When Prescribing Inhalers. *J Am Board Fam Med* 2023; **36:** 356–59. https://doi.org/10.3122/jabfm.2022.220292R2.

12 King LP, Brown J. Educating patients as medicine goes green. *Virtual Mentor* 2009; **11:** 427–33. https://doi.org/10.1001/virtualmentor.2009.11.6.ccas1-0906.

13 Müller F, Skok JI, Arnetz JE, Bouthillier MJ, Holman HT. Primary Care Clinicians' Attitude, Knowledge, and Willingness to Address Climate Change in Shared Decision-Making. *J Am Board Fam Med* 2023. https://doi.org/10.3122/jabfm.2023.230027R1.

14 Parker J. Environmentally friendly inhalers: issues for the general practice consultation. *Br J Gen Pract* 2022; **72:** 484–85. https://doi.org/10.3399/bjgp22X720821.

15 Parker J. Barriers to green inhaler prescribing: ethical issues in environmentally sustainable clinical practice. *J Med Ethics* 2023; **49:** 92–98. https://doi.org/10.1136/jme-2022-108388.

16 Parker J. The scope of patient, healthcare professional and healthcare systems responsibilities to reduce the carbon footprint of inhalers: a response to commentaries. *J Med Ethics* 2023; **49:** 187–88. https://doi.org/10.1136/jme-2023-108908.

17 Resnik DB, Pugh J. Green bioethics, patient autonomy and informed consent in healthcare. *J Med Ethics* 2023. https://doi.org/10.1136/jme-2023-109404.

18 Richie C. "Green informed consent" in the classroom, clinic, and consultation room. *Med Health Care Philos* 2023; **26:** 507–15. https://doi.org/10.1007/s11019-023-10163-x.

19 Rieder TN. Green prescribing is good, but patients do not have a duty to accept it. *J Med Ethics* 2023; **49:** 104–05. https://doi.org/10.1136/jme-2022-108846.
20 Segers S, Proost M de. Complication for a greener medical ethics code: assisted reproduction. *J Med Ethics* 2023. https://doi.org/10.1136/jme-2023-109667.
21 van Gils-Schmidt HJ, Salloch S. Physicians' duty to climate protection as an expression of their professional identity: a defence from Korsgaard's neo-Kantian moral framework. *J Med Ethics* 2023. https://doi.org/10.1136/jme-2023-109203.
22 Wabnitz K-J, Gabrysch S, Guinto R, et al. A pledge for planetary health to unite health professionals in the Anthropocene. *Lancet* 2020; **396:** 1471–73. https://doi.org/10.1016/S0140-6736(20)32039-0.
23 Wiesing U. Climate change and the different roles of physicians: a critical response to "A Planetary Health Pledge for Health Professionals in the Anthropocene". *Med Health Care Philos* 2022; **25:** 161–64. https://doi.org/10.1007/s11019-021-10051-2.
