# Supplementary material for: Should healthcare professionals include aspects of environmental sustainability in clinical decision-making? A systematic review of reasons
Source: BMC Med Ethics. 2025 Jul 3;26:78. doi: 10.1186/s12910-025-01230-4 (PMC12226885; doi:10.1186/s12910-025-01230-4)
Supplement: Supplementary file 2 — Supplementary Material 2 [file 12910_2025_1230_MOESM3_ESM.pdf]

Supplement 2: Meta data of the included articles

Article name	1st Author Name	1st author affiliation	1st author's specialisation / profession	Journal name	Type of Text indicated by Journal	Type of Text clustered by authors	Language	Profession addressed	Year of Publication	Topic	Review method
Talking about Climate Change and Environmental Degradation with Patients in Primary Care: A Cross-Sectional Survey on Knowledge, Potential Domains of Action and Points of View of General Practitioners	André, Hélène	Department of Family Medicine, Center for Primary Care and Public Health (Unisanté), University of Lausanne, 1004 Lausanne, Switzerland	Research Fellow	International Journal of Environmental Research and Public Health	Article	Quantitative data analysis	English	GPs, Global Health Professionals	2022	General; Reprod. medicine	peer reviewed
Dual duties to patient and planet: time to revisit the ethical foundations of healthcare?	Bhopal, Anand	Department of Global Public Health and Primary Care, University of Bergen, Bergen, Norway	PhD Candidate	Journal of Medical Ethics	Commentary	Commentary	English	Bioethicists; Physicians	2023	Inhaler; General	internally peer reviewed
What Are Risks and Benefits of Not Incorporating Information about Population Growth and Its Impact on Climate Change into Reproductive Care?	Brown, Benjamin P.	Department of Obstetrics and Gynecology, University of Chicago	Gynaecology	AMA Journal of Ethics	Commentary	Case presentation	English	Gynaecologists	2017	Reprod. medicine	peer reviewed
Doctors and Climate Change: Impact of Medical Ethics	Chambers, John C.	Macmillan consultant and medical director Katharine House Hospice, Adderbury, Oxfordshire	Medical Director	BMJ	Letter	Argumentative Text	English	Physicians	2008	General	not peer reviewed
Harmonising green informed consent with autonomous clinical decision-making: a reply to Resnik and Pugh	Cohen, Eva Sayone	Department of Obstetrics and Gynaecology, Amsterdam UMC, Vrije Universiteit, Amsterdam, the Netherlands	PhD candidate; Physician	Journal of Medical Ethics	Response	Commentary	English	Bioethicists; Physicians	2024	General	internally peer reviewed
Green inhaler prescribing and the ethical obligations of physicians	Coverdale, John	Menninger Department of Psychiatry and Behavioral Science and Center for Ethics, Baylor College of Medicine, Houston, Texas, USA	Professor/Physician	Journal of Medical Ethics	Commentary	Commentary	English	Bioethicists; Physicians	2023	Inhaler	internally peer reviewed

Supplement 2: Meta data of the included articles

Lachgas-Analgesie unter dem Aspekt: Nachhaltigkeit im Kreißsaal	Dresen, Franziska	Hochschule für Gesundheit Bochum Department für Angewandte Gesund-heitswissenschaften Studienbereich Heb-ammenwissenschaft Gesundheitscampus 6–8 44801 Bochum Deutschland	Midwife; Research Fellow	Hebamme	Research Article	Argumentative Text	German	Midwives; Gynaecologists	2023	Gynaecology	not peer reviewed
'Green' bioethics widens the scope of eligible values and overrides patient demand: comment on Parker	Herlitz, Anders	Department of Philosophy, Linguistics & Theory of Science, University of Gothenburg, Goteborg, Sweden	Research Fellow; Associate Professor	Journal of Medical Ethics	Commentary	Commentary	English	Bioethicists; Physicians	2023	Inhaler	internally peer reviewed
Nachhaltiges Verordnungsverhalten und Implementierung in Leitlinien	Heuer, Ruben	Division of Evidence-Based Medicine, Klinik für Dermatologie, Venerologie und Allergologie, Charité – Universitätsmedizin Berlin, Gemeinsame Einrichtung der Freien Universität Berlin, Humboldt-Universität zu Berlin und dem Berlin Institute of Health, Berlin, Deutschland	Research Fellow	Die Dermatologie	Article	Argumentative Text	German	Dermatologists	2023	Dermatology; General	peer reviewed
Thinking "Green" When Treating "Pink Puffers" and "Blue Bloaters"—Reducing Carbon Footprint When Prescribing Inhalers	Holman, Harland T.	Department of Family Medicine, Michigan State University, Grand Rapids, MI, USA	Assistant Professor; Clinician	Journal of the American Board of Family Medicine	Special Communication	Informative Text	English	GPs	2023	General	externally peer reviewed
Educating Patients as Medicine Goes Green	King, Louise P.	Parkland Memorial Hospital in Dallas	Assistant Professor of Obstetrics, Gynaecology and Reproductive Biology	AMA Journal of Ethics	Commentary	Case presentation	English	Gynaecologists	2009	Gynaecology; general	internally peer reviewed

Supplement 2: Meta data of the included articles

Primary Care Clinicians' Attitude, Knowledge, and Willingness to Address Climate Change in Shared Decision-Making	Müller, Frank	Department of Family Medicine, Michigan State University, 15 Michigan St NE, Grand Rapids MI 49503	Adjunct Professor; Clinician Researcher	Journal of the American Board of Family Medicine	Research Article	Quantitative data analysis	English	GPs	2023	General	externally peer reviewed
Barriers to green inhaler prescribing: ethical issues in environmentally sustainable clinical practice	Parker, Joshua	Faculty of Health and Medicine, Lancaster Medical School, Lancaster University, Lancaster, UK	PhD Candidate; GP	Journal of Medical Ethics	Feature Article	Argumentative Text	English	Physicians	2022	Inhaler	peer reviewed
Environmentally friendly inhalers: issues for the general practice consultation	Parker, Joshua	Faculty of Health and Medicine, Lancaster Medical School, Lancaster University, Lancaster, UK	PhD Candidate; GP	British Journal of General Practice	Informative Article	Informative Text	English	GPs	2022	Inhaler	peer reviewed
The scope of patients, healthcare professionals and healthcare systems responsibilities to reduce the carbon footprint of inhalers: a response to commentaries	Parker, Joshua	Faculty of Health and Medicine, Lancaster Medical School, Lancaster University, Lancaster, UK	PhD Candidate; GP	Journal of Medical Ethics	Commentary	Commentary	English	Bioethicists; Physicians	2023	Inhaler	internally peer reviewed
Green Bioethics, Patient Autonomy, and Informed Consent in Health Care	Resnik, David B.	National Institute of Environmental Health Sciences, Durham, North Carolina, USA	Bioethicist	Journal of Medical Ethics	Research Article	Argumentative Text	English	Bioethicists; Physicians	2023	General	peer reviewed
"Green informed consent" in the classroom, clinic, and consultation room	Richie, Christina	Ethics of Technology, Edinburgh Futures Institute, The University of Edinburgh, Edinburgh, Scotland	Bioethicist	Medicine, Health Care and Philosophy	Scientific Contribution	Argumentative Text	English	Bioethicists; Physicians	2023	General	peer reviewed
Green prescribing is good, but patients do not have a duty to accept it	Rieder, Travis N.	Berman Institute of Bioethics, Johns Hopkins University, Baltimore, Maryland, USA	Associate Research Professor	Journal of Medical Ethics	Commentary	Commentary	English	Bioethicists; Physicians	2023	Inhaler	internally peer reviewed
Complication for a greener medical ethics code: assisted reproduction	Segers, Seppe	Department of Philosophy and Moral Sciences, Ghent University, Gent, Belgium	Bioethicist	Journal of Medical Ethics	Commentary	Commentary	English	Bioethicists; Physicians	2023	Reprod. medicine	internally peer reviewed
Green bioethics	ten Have, Henk	Duquesne University, Pittsburgh, USA	Professor/ Physician	Medicine, Health Care and Philosophy	Editorial	Editorial	English	Bioethicists; Physicians	2023	General	internally peer reviewed

Supplement 2: Meta data of the included articles

Physicians' duty to climate protection as an expression of their professional identity: a defence from Korsgaard's neo-Kantian moral framework	van Gils-Schmidt, Henk Jasper	Department Health Sciences, Hamburg University of Applied Sciences, Hamburg, Hamburg, Germany	Research Fellow	Journal of Medical Ethics	Feature Article	Argumentative Text	English	Bioethicists; Physicians	2023	Professionalism; General	peer reviewed
A pledge for planetary health to unite health professionals in the Anthropocene	Wabnitz, Katharina-Jacqueline	Department of Public Health and Primary Care, The Primary Care Unit, University of Cambridge CB2 0SR, UK	Research Fellow	The Lancet	Commentary	Argumentative Text	English	Physicians	2020	Professionalism; General	not peer reviewed
Climate change and the different roles of physicians: a critical response to "A Planetary Health Pledge for Health Professionals in the Anthropocene"	Wiesing, Urban	Institute for Ethics and History of Medicine, University of Tübingen, Gartenstr. 47, 72074 Tübingen, Germany	Medical Ethicist	Medicine, Health Care and Philosophy	Short Communication	Argumentative Text	English	Bioethicists; Physicians	2021	Professionalism; General	internally peer reviewed
