# Supplementary material for: Should healthcare professionals include aspects of environmental sustainability in clinical decision-making? A systematic review of reasons
Source: BMC Med Ethics. 2025 Jul 3;26:78. doi: 10.1186/s12910-025-01230-4 (PMC12226885; doi:10.1186/s12910-025-01230-4)
Supplement: Supplementary file 3 — Supplementary Material 3 [file 12910_2025_1230_MOESM4_ESM.pdf]

Table of reasons

List of abbreviations:
(+) = positive reason, for implementing aspects of environmental sustainability in clinical decision-making
(-) = negative reason, against implementing aspects of environmental sustainability in clinical decision-making
(+/-) = ambivalent reason regarding implementing aspects of environmental sustainability in clinical decision-making
The frequency of the reason identified in the publication is indicated after publication number (for example: Publication [14] (3x)).
If no number of reasons is indicated, the reason was identified once in the named publication.
The publication number refers to the list of articles included in the review (see Supplement 1).

Main category	Coding rule	Subcategory (if available)	Reason	Specifications	Example	Publication(s)
Respect for patient autonomy	This code is used when authors use respect for autonomy as a reason for or against implementing aspects of environmental sustainability in health counselling of climate-damaging treatments.<br>Definition:<br>Three-condition theory according to		Origin of respect for patient autonomy (+)	Reason mentions the historical origin of patient autonomy and focuses on the original definition	"(…) [R]espect for patient autonomy was originally formulated as a negative right—do not impose medical treatments—constructed in part as a response to the medical crimes and violations of human rights in medicine of the 20th century, including the Tuskegee Syphilis Study, human experimentation in Nazi concentration camps and the Henrietta Lacks case. They propagate a misunderstood version of 'patient autonomy', currently plaguing many parts of the Global North. As used here, and in many places, patient autonomy has shifted to an entitled disposition on the part of the medical consumer. From that consumeristic mindset, green information may be inappropriate; if a person goes to buy a car, conversations about environmental impact may be unwelcome. But patients should not be regarded as consumers, nor is healthcare a	[5]

	Beauchamp and Childress:<br>(1) Intentionality: Correspondence to the actor's conception of the act in question. Acknowledges conflicting wants and desires as well as undesired but foreseen outcomes.<br>→ No degree<br>(2) Understanding: Substantial degree of understanding the action in question.<br>→ Degree is certain<br>(3) Noncontrol: A person is free of controls exerted either by external sources (more important for autonomy) or internal states (less				capital service." (5)	
			Morals are not relevant for patient autonomy (+/-)		"Moreover, what is morally right or wrong for patients to choose is normally not seen as relevant for the issue of whether or not their autonomy should be respected." (9)	[9]
			Reproductive medicine debate: Serious strain on patient autonomy (-)		"(…) [W]here even 'mere' dissuasion may be considered a serious strain on autonomy, and an unjust targeting of people who cannot reproduce otherwise than through ART [assisted reproductive technology] (…)." (20)	[20]
			Overriding patient's refusal is threat to doctor's trust (-)		"(…) [I]f informed consent is important for trust, then failing to respect a patient's refusal simply undermines the distinct contribution informed consent makes to trust." (15)	[15]
		Informed consent	Educating patients is part of "going green" (+)		"That said, educating patients on the need for multiple-use equipment and addressing their concerns regarding safety is an important part of the process of "going green." Perhaps in some instances, merely offering patients a handout that details the problem with medical waste and the process and safety of sterilization will suffice." (12)	[12]
			Physicians would	Physicians would otherwise	Even if this data is unknown, consenting the patient about the side efects [sic!] of their	[3], [5], [18]

	important for autonomy)<br><br>→ Degree is certain<br><br>To code:<br><br>- All reasons, no matter what their conclusion is, as long as they fit the above-mentioned definition of autonomy<br>- All reasons regarding the concept of informed consent<br>- All reasons regarding the concept of shared decision-		withhold information (+)	paternalistically withhold some information that could be of patient's interest (e.g., information about the climate impact of a healthcare measure)	care—which may include climate change health hazards—would fulfl [sic!] the obligation of the physician for truth-telling and give the patient relevant information with which to make a treatment choice." (18)	(2x)
			Patients should know about climate impact of healthcare measures (+)		"While patients should be aware that their health care emits carbon, contributes to climate change, and may affect them through climate change health hazards, they should not bear the burden of research into the environmental effects of health care (…). Therefore, patients may expect—and be prepared— for health care providers to disclose the environmental effect of various procedures during green informed consent, which is already the standard in some health care facilities." (18)	[18]
			No need for specific information if both options are standard (-)	The patient does not need to be given additional information if both treatment alternatives are equivalent	"Both pieces of equipment are considered standard of care, and a strong argument can be made that a patient need not be informed of each piece of equipment that will be used for treatment." (12)	[12]

	making<br>Not to code:<br>- Reasons not fitting this definition of patient autonomy		Physicians should act in patients' interest (-)		"The most important medical role is the one towards a patient. In this relationship, a physician is to a large extent relieved of further social responsibilities and should act primarily in a way that is in the patient's best interest." (23)	[23]
		Shared decision-making	Eliciting patient's wishes/values to form a decision (+)		„Das Wissen über die mangelnde Wirksamkeit, die möglichen Nebenwirkungen ebenso wie die klima- und ozonschädigenden Effekte von N2O sind werdenden Müttern zur Verfügung zu stellen. Hierzu bieten sich v. a. Vorsorgekontakte in der Schwangerschaft und Geburtsvorbereitungskurse an. Um Frauen in ihrer Entscheidungsfindung hinsichtlich der Schmerzbewältigung während der Geburt bestmöglich zu stärken, sollten sie ausführlich und klimasensibel über die verschiedenen, evidenzbasierten Methoden der Schmerztherapie informiert werden." (7)	[3] (2x), [5], [7], [15]
			Patients seem to be willing to hear climate impact of healthcare measures (+)		"(…) [P]atients appear willing to incorporate climate considerations in medicine. A study by Reismann et al showed that 71% of patients visiting a general practitioner or gynaecologist in Germany indicated a willingness to engage in climate-friendly behaviour if physicians informed them about climate-related health risks.[7] Wilkinson and Woodcock argue that 'doctors, nurses and pharmacists need information on the carbon footprint of inhalers to be able to provide that data to patients in a spirit of partnership in taking treatment	[5]

					decisions. Patients do care about the carbon footprint of their treatment' and offer three studies to back up their statement.[8]" (5)	
			Unclear, what practising sustainable healthcare really means (+/-)		"It is unclear what the duty of practicing sustainable healthcare means at the level of doctor patient interaction." (20)	[20]
			Only adequate when patient expresses interest (+/-)	Mentioning the climate impact of healthcare measures in the consultation is only adequate if the patients explicitly wishes for the information	"We agree that disclosure of information pertaining to the environmental impacts of treatments could promote autonomous decision-making in situations where patients have clearly expressed concerns regarding the environment." (17)	[17]
			It is important how the conversation occurs (+/-)		"While patients are receptive to change for environmental considerations, changes to the management of a chronic condition can be distressing. How these conversations occur is important in moving towards more environmentally sustainable respiratory care, and sensitive, patient-centred communication will underpin making decisions that are good for the patient and good for the planet." (15)	[15]
			Sometimes,		"In such circumstances [having two clinically	[17]

			patients should not have a choice (+/-)		equivalent interventions with significantly different environmental impacts], there could be compelling moral reasons to not give patients a choice between these treatment alternatives, assuming that there are no other factors that would weigh against this decision.”	
			Decisions should be made collaboratively (+/-)		“Although there is a strong case for prescribing DPIs rather than MDIs for climate change reasons, implementation of the recommendation should not be automatic but should take into account patient preference and willingness and ability to use DPIs safely. (…). From a practical point of view, changing an ongoing and efficient inhaler therapy should be well considered, weighing the pros and cons, and always discussed with the patient in the context of shared decision-making.” (11)	[10], [11]
			Physicians should not impose their values on a patient (-)		“It is not appropriate for Dr. Stuart to impose environmental protection values on Emily’s reproductive decision making, as this risks undermining her autonomy as well as perpetuating injustice.” (3)	[3]
			Autonomy reigns supreme if a patient is steadfast (-)		“It may be that some reject a non-propellant inhaler and would prefer their old ‘puffer’. The clinician’s response to this situation is well established: explore the patient’s perspective, correct misunderstandings, and try and persuade them to change. But, if the patient is	[14]

					steadfast, autonomy reigns supreme." (14)	
			Danger of damage to the patient-provider relationship (-)		"However, we are skeptical [sic!] of the claim that there is a generalizable legal or ethical duty to disclose environmental impact information to all patients. We shall argue that providers (e.g., physicians, nurses, and pharmacists) should not routinely disclose information about the environmental impacts of treatments during the consent process because this may subvert patients' autonomous decision-making and damage the patient-provider relationship." (17)	[17]
			Physicians feel constrained (-)		"(…) [I]t is questionable whether health professionals will actually raise this issue in daily care settings. The majority of professionals is well aware that climate change is happening and is caused by humans. Nonetheless, they feel constrained when discussing and advocating climate change as a human health issue (Kotcher et al. 2021). They prefer other options (…). Given this background it is doubtful whether health professionals will decarbonize their own practices of patient care." (8)	[8]
			GPs haven't been approached by patients (-)		"As half of all clinicians reported having never been approached by patients concerned about climate change, it is likely that climate change has not become a topic at their clinics." (13)	[13]
Respect for	This code is used		There is no	Autonomy stands	"However, we will claim that it is only radical in	[2]

patient autonomy – transformed	when the reason mentions patient autonomy, but puts the emphasis not on the individual but broader.		vacuum around autonomy (+)	in tension with other foundational ethics concerns	so far as one perceives (A) patient autonomy and healthcare entitlements as existing within a vacuum, unrelated to other foundational ethics concerns (…).” (2)	
			Autonomy only as long as no other person is harmed (+)		“The primary focus of ethical decision making in medicine is the patient and his or her immediate environment. Beneficence, non-maleficence, justice, and dignity are subservient to autonomy. Good of the community is obscured within the principle of justice. However, when self interest and inadequate resources harm others, autonomy loses integrity.” (4)	[4]
Non-Maleficence	This code is used when authors use non-maleficence as a reason for or against implementing aspects of environmental sustainability in health counselling of climate-damaging treatments.		Avoidance of overdiagnosis or over-treatment (+)		“However, not all health care is medically necessary or clinically indicated. Much has been written about medical waste, the harm of overtreating, and medicalization (Barratt and McGain 2021). Since clinical necessity is a prerequisite for clinical medicine, in addition to green informed consent for medically necessary procedures, aspects of health care delivery outside that scope can be immediately targeted for reduction with environmental impact as an additional reason alongside sound economic and biomedical arguments (Edoka and Stacey 2020).” (18)	[18]
	Definition according to Beauchamp and		Maintain trust/protect patient’s		“As a pro tanto obligation, the need to minimise expected harm can, for the doctor, be over-ridden by a need to maintain trust or to protect	[15]

	Childress: To abstain from causing harm to others. To not inflict evil or harm. → Intentional avoidance of actions		health to do no harm (-)		their patient's health." (15)	
			Doing harm in not treating patients optimally (-)		"However, if the best intervention is not chosen for environmental reasons and the patient is not treated optimally and worse off, then there is a serious conflict." (23)	[23]
Non-maleficence – transformed	This code is used when the reason mentions non-maleficence or the duty to do no harm but widens the scope of it in relation to the climate crisis		Connecting healthcare delivery with the necessity to reduce GHG emissions (+)		"(…) [D]octors and health care professionals must connect their health care delivery with carbon emissions and minimize resource use when possible as a part of their obligation to do no harm (the principle of non-maleficence). Patients receiving treatments should be aware that their health care in the clinic may lead to more medical treatments later, should they encounter climate change health hazards." (18)	[7], [8], [18]
			Duty to avoid expected harm (+)		"Even if MDIs do not directly cause harm, their significant global warming potential increases the risk of climate-mediated harms and so in this way is associated with expected harm. The question of how patients and practitioners ought to respond to the emissions of inhalers is important and cannot simply be dismissed as making no difference. Moreover, since there is another easily available alternative (DPIs) then it is pro tanto wrong to use an MDI. For some patients, a DPI is not clinically appropriate, so even though MDIs increase expected harm, a	[14], [15] (2x)

					DPI is not an easily available alternative for the patient who is unable to use one." (15)	
Beneficence	This code is used when the authors use beneficence as a reason for or against implementing aspects of environmental sustainability in health counselling of climate-damaging treatments.<br>Definition according to Beauchamp and Childress: Rules of beneficence (1) present prositive requirements of action, (2) need not always be be followerd impartially and (3) generally do not provide reasons for legal punishment when agents fail to		Co-benefits of "green prescribing" (+)		"Ideally, the patient [who is overusing short-acting beta agonists] would start a DPI preventer; however, any preventer including an MDI will still have a smaller carbon footprint. Starting a preventer therapy is so clearly in the patient's interests that if an MDI is preferred, what is in the patient's interests shapes what options are available to minimise expected harm. It should be apparent that there are various ways of minimising expected harm within the confines of maintaining trust and acting in the patient's interests and the principle of environmental prescribing is useful in guiding this." (15)	[15]
			Help patients to find out what they want (+)		"(…) [H]elping Emily explore and resolve the emotional distress that she is experiencing as she considers the potential environmental impact of a subsequent pregnancy and whether it is acceptable to bring a new child into the world at this time is in accordance with the principle of beneficence." (3)	[3]

	abide by them.					
Justice	This code is used when authors use justice according to Beauchamp and Childress as a reason for or against implementing aspects of environmental sustainability in health counselling of climate-damaging treatments.		Higher cost to reduce GHG emissions but no direct difference for patients (+/-)		"We might ask whether it is fair for a healthcare system to accept higher costs to reduce its carbon footprint if this makes no direct difference to patients locally, instead producing spatially and temporally distributed benefits. This raises a question of distributive justice regarding the fair share of the benefits and burdens of tackling climate change for a publicly funded healthcare system." (15)	[15]
			Possibility of perpetuating injustice due to counselling (-)		"It is not appropriate for Dr. Stuart to impose environmental protection values on Emily's reproductive decision making, as this risks undermining her autonomy as well as perpetuating injustice." (3)	[3]
Environmental justice (justice transformed)	This code is used when the reason mentions the concept of environmental justice and does not focus necessarily only on distributive justice with the focus of an individual patient		Duty to accept higher costs due to wider health benefits for low or middle income countries (+)		"Nonetheless, there may be wider health benefits. (…). However, most of these benefits will be felt in low-income and middle-income countries. Any health benefits of climate change mitigation for the UK are likely to occur long after inhalers are changed." (15)	[15]
			Conservation of good living standards for everyone (+)		"A core objective is to transform human values, behaviours, and societal structures to maintain the "safe and just operating space for humanity" we depend on to thrive." (22)	[22]

			Acknowledging local public health responsibility/ global health responsibility (+)		"By incorporating environmental considerations into the informed consentvprocess, healthcare providers contribute to a more just and equitable distribution of the environmental burdens associated with medical treatments. Acknowledging and addressing the environmental impact of healthcare interventions also aligns with the broader concept of global public health responsibility." (5)	[2], [4], [5], [18], [21]
			Health consequences address all affected parties (+)		"(…) [W]e will claim that it is only radical in so far as one perceives (…) the health consequences of a given healthcare budget are limited solely to the designated recipients rather than all affected parties." (2)	[2]
			Patients save GHG emissions for doctors to fly around the world to attend conferences (-)		"(…) [T]hink about what we would be demanding of patients in the context of how big their contribution to climate change is. So consider: if my physician attends one international conference in a year, flying from London to Los Angeles, that round trip flight alone increases their carbon footprint by about 879kg (double that for premium seating).[8] So doing the math, taking a relatively routine, economy class flight would wipe out the GHG savings of about 31 MDI inhalers. While many of us do feel some moral pressure to reduce the amount we fly, the freedom of the more privileged in society to fly anyway sits uncomfortably with the control that prescribing	[19]

					physicians have over their patients." (19)	
		Polluter pays	Whoever causes harm needs to fix the problem (+)		"Polluter pays' is a straightforward principle that states that those causing global warming should pay the costs of fixing the problem. Henry Shue explains the reasoning behind this: All over the world parents teach their children to clean up their own mess… If whoever makes a mess receives the benefits and does not pay the costs, not only does he have no incentive to avoid making as many messes as he likes, but he is also unfair to whoever does pay the costs." (15)	[15]
			Who exactly is the polluter? Difficult to find out (-)		"(…) [I]t is not entirely obvious that the NHS is the polluter. Perhaps it is, but plausibly it could also be the patient using the MDI, the prescriber, the pharmaceutical company and so forth. If we are not convinced that the NHS is the polluter, it may be considered unfair that the NHS pays. Worse still, it is difficult to make the NHS as an institution pay because the funds for more expensive inhalers must be found either through the taxpayer or from elsewhere within the NHS. As it is patients or taxpayers who ultimately pay, we may be especially concerned by a disconnect between who the polluter is and who pays. (15)	[15]
			Subsistence emissions are		"Some claim that 'subsistence' as opposed to 'luxury' emissions are an exception to a polluter pays principle. Shue argues that to treat all	[15]

			exempt (-)		emissions as equal regardless of their purpose is to 'ignore the fact that some sources [of greenhouse gas emissions] are essential and even urgent for the fulfilment of vital needs and other sources are inessential or even frivolous'.[36]" (15)	
Politicisation	This code is used when authors refer to politicisation as a reason for or against implementing aspects of environmental sustainability in the consultation practise.		Discussion makes patients aware of the climate crisis (+)		"(…) [A] discussion with patients on the effects of various forms of inhalers on the environment may bring to their attention the seriousness of anthropogenic climate change and open opportunities for patients to consider other ways in which they can protect the environment." (5)	[5] (2x)
			Politicisation is no unusual phenomenon for clinicians (+)		"First, in clinical practice, healthcare providers routinely navigate through a spectrum of diverse and potentially sensitive topics, some of which may be perceived as equally or even more 'politicized' than climate change. Examples vary by country (…). Physicians are accustomed to addressing these challenging subjects and receive extensive training at communication in healthcare provision.[3] Even if a topic is perceived as politicised, physicians maintain a prima facie obligation to engage individuals in discussions about health risks. Besides, bioethicists and clinicians should refrain from assuming that climate change is universally politicised to the same degree." (5)	[5]

			Reproductive issues are too sensitive and controversial (-)		“Not surprisingly, GPs were reluctant to discuss reproductive health with patients (to limit unwanted pregnancies and population growth). This is indeed a very sensitive and controversial topic, raising important ethical issues that have a long political history rather than health implications [33].” (1)	[1]
Reasons addressing levels of action	This code is given whenever the reason addressed in the publication refers to a level of action, e.g. the individual level, the systemic level, micro/meso/macro level etc.		Only addressing the meta level does not solve all the problems (+)		“If a patient has well-controlled asthma, say with a Fostair 100/6 MDI device, and then Herlitz et al remove this from the formulary, how do doctors go about switching? Do doctors just go ahead and prescribe the dry powered equivalent without consultation? Do they discuss this with the patient? If they do, what should they discuss? How should the clinician respond to the patient who doesn’t want to switch or who is concerned that their control should worsen? I can see the attractiveness of taking an institutional or structural approach to the problem of inhalers but it is myopic to think this can be divorced from the interactions that doctors have with their patients. Indeed, I came to this issue because of challenges I faced in my own practice.” (16)	[16]
			Pharmaceutica l/chemical products as biggest contributor to climate crisis		“In order to reach this goal, health professionals should discuss with patients medical treatment options including their environmental risks and potential contributions to climate change. This is a reasonable proposal since pharmaceutical and chemical products rather than buildings	[8]

			within healthcare sector (+)		and transport seem to be the biggest contributors to the environmental footprint of the healthcare sector (Steenmeijer et al. 2022).” (8)	
			The focus on the individual weakens argumentation (-)		“Parker’s focus on the individual clinical choice situation rather than the institutional one weakens the attractiveness of his move towards ‘green’ bioethics, as it maintains these limitations, while the ground for moving towards ‘green’ bioethics activates criticism of them. For instance, two authors of this response have recently presented principles and frameworks for ascribing value to environmental sustainability when evaluating healthcare practices.” (9)	[9]
			It is not the physicians’ task to decide (but society’s) (-)		“(…). These are decisions that need to be clarifed at the societal level.” (23)	[23]
			Providers should focus on systems-level approaches (-)		“Providers should focus on advocating for system-level changes in health care financing, organization, and delivery whilst using discretion when bringing up environmental concerns with their patients.” (17)	[17]
Professionalis m	This code is given when the reason addresses some	Healthcare professionals ’ role in	“Reasonable people” care about the		“(…) ‘[R]easonable people’ care about environmental damage. Healthcare professionals are often included in this category	[5]

	sort of professionalism, either in the form of healthcare professionals' roles or in the form of professions' codes or initiatives	general	climate crisis, healthcare professionals are included in that group (+)		of 'reasonable people'." (5)	
			Ethical imperative of planetary health principles (+)		"Rooting planetary health principles in the professional ethos, education, and practice of all health professionals is imperative.[12]" (22)	[22]
		Physicians' role	Knowing and teaching climate protection is medical expertise (+)		"Here, we test the claim that our responsibility for promoting patient autonomy and avoiding harm coexists with our responsibilities towards the planet and one another, independently of national borders. This leads to a radically different starting point for considering the implications of the climate crisis for medical ethics and priority setting." (2)	[2], [6] (2x), [10] (3x), [21] (3x)
			Trust is built while disclosing climate-related information (+)		"(…) [T]ransparent communication builds trust between patients and healthcare providers. By openly addressing environmental concerns, healthcare providers can strengthen the patient–provider relationship and demonstrate a commitment to honesty, thereby fostering a culture of trust that extends beyond individual consultations." (5)	[5], [6]
			Meddled responsibilities		"Especially the responsibilities in environmental protection are difficult to define. The polluters	[23]

			(-)		are often not confronted with the consequences and the results of the environmental<br><br>changes show up only in later generations. It is difficult to determine exactly how much an individual action contributes to environmental harm (see e.g. Wardrope 2020). In view of this complexity, it is even more important to establish the clear responsibility (…).” (23)	
			Patient’s wish comes first (-)		“If conflicts were to arise with other roles of the physician, the role towards a patient mostly has precedence.“ (23)	[23]
			Physicians’ obligation to confidentiality (-)		“If a physician discovers within the course of treatment, that a patient is violating environmental protection regulations and intends to do so in the future, should the physician report this to the police or environmental authorities? Thus far, confidentiality prohibits such behavior for a good reason mentioned above: trust.” (23)	[23]
			Trust might be lost (-)		“But should physicians have to decide personally in the physician–patient relationship whether to help a patient or reduce greenhouse gas emissions? No, physicians must be relieved of such decisions in the physician–patient relationship, otherwise trust in the medical profession will be lost.” (23)	[23]

			Important ethical decisions must be made outside of the physician-patient relationship (-)		“(…) [I]mportant ethical decisions, which are related to society, must be made outside of the physician–patient relationship.” (23)	[23]
		Professions’ codes / initiatives	Physicians’ codes already recommend practising in environmentally conscious ways (+)		“This emphasis on environmental sustainability is in line with popular discourse as well growing scholarly attention in medical ethics for healthcare’s contribution to climate change. Recent research analyses, for instance, the ‘greening’ of informed consent and related bioethical principles (2,3)” (20)	[20], [21]
			Changing codes might be seen as normatively binding (+)		“(…) [C]hanges in the ethical codices that guide the medical profession can be understood as normatively binding for the medical professional.” (21)	[21]
			Physicians hold responsibility over a single patient (-)		“Efforts to redefine the roles and responsibilities of physicians in regard to climate change and environmental degradation, such as the Planetary Health Pledge for Health Professionals in the Anthropocene have been severely criticized because they compromise the primary responsibility of the physician who is acting in the best interest of the individual	[8], [23]

					patient (Wiesing 2022).“ (8)	
Other	This code is given when the reason found in the publications does not fit any other inductive or deductive main category		Epistemic openness (+)		“I will acknowledge and respect diverse sources of knowledge and knowing regarding individual, community, and planetary health such as from Indigenous traditional knowledge systems while challenging attempts at spreading disinformation that can undermine planetary health.” (22)	[22]
			Complexity of cause-effect relationship of climate crisis (+/-)		“So sehr jedoch ein verantwortungsvoller Umgang mit folgeträchtigen medizinischen Entscheidungen geboten ist, so diffizil stellt sich dessen Umsetzung im klinischen Alltag dar. Die Komplexität klimatischer Ursache-Wirkung-Beziehungen scheint eindeutige Aussagen bezüglich konkreter Einzelfallentscheidungen nahezu unmöglich zu machen.“ (10)	[10]
			Dependence on topic of discussion in consultation (+/-)		“Instead of blanketly rejecting routine environmental disclosure, it is important to not collapse important differences in clinical specialties and clinical significance of medical decisions. For instance, it might be less appropriate to bring up the environmental impact of chemotherapy and more relevant for asthma inhalers or contraception.” (5)	[5]
			Ethical conflicts between individuals and		“Hinzu kommen offensichtliche ethische Konflikte zwischen den Bedürfnissen individueller Patient*innen und denen einer abstrakten Allgemeinheit.“ (10)	[10]

			public (+/-)			
			Technical difficulties quantifying emissions (-)		"The relative value of carbon emissions in this revised equation would, in turn, be contingent on many other ethical factors, including risk aversion (since the harms of a given quantity of emissions are uncertain), discount rates (since the negative impacts are concentrated in the future) and whether the population of concern is national or global (since climate impacts are largely imposed on people far away). This is not only difficult to quantify[7] but value laden and morally complex to implement in practice.[8] [9]" (2)	[2]
