# Supplementary figures and images for: Should healthcare professionals include aspects of environmental sustainability in clinical decision-making? A systematic review of reasons
Source: BMC Med Ethics. 2025 Jul 3;26:78. doi: 10.1186/s12910-025-01230-4 (PMC12226885; doi:10.1186/s12910-025-01230-4)

# Supplement 4: Code tree

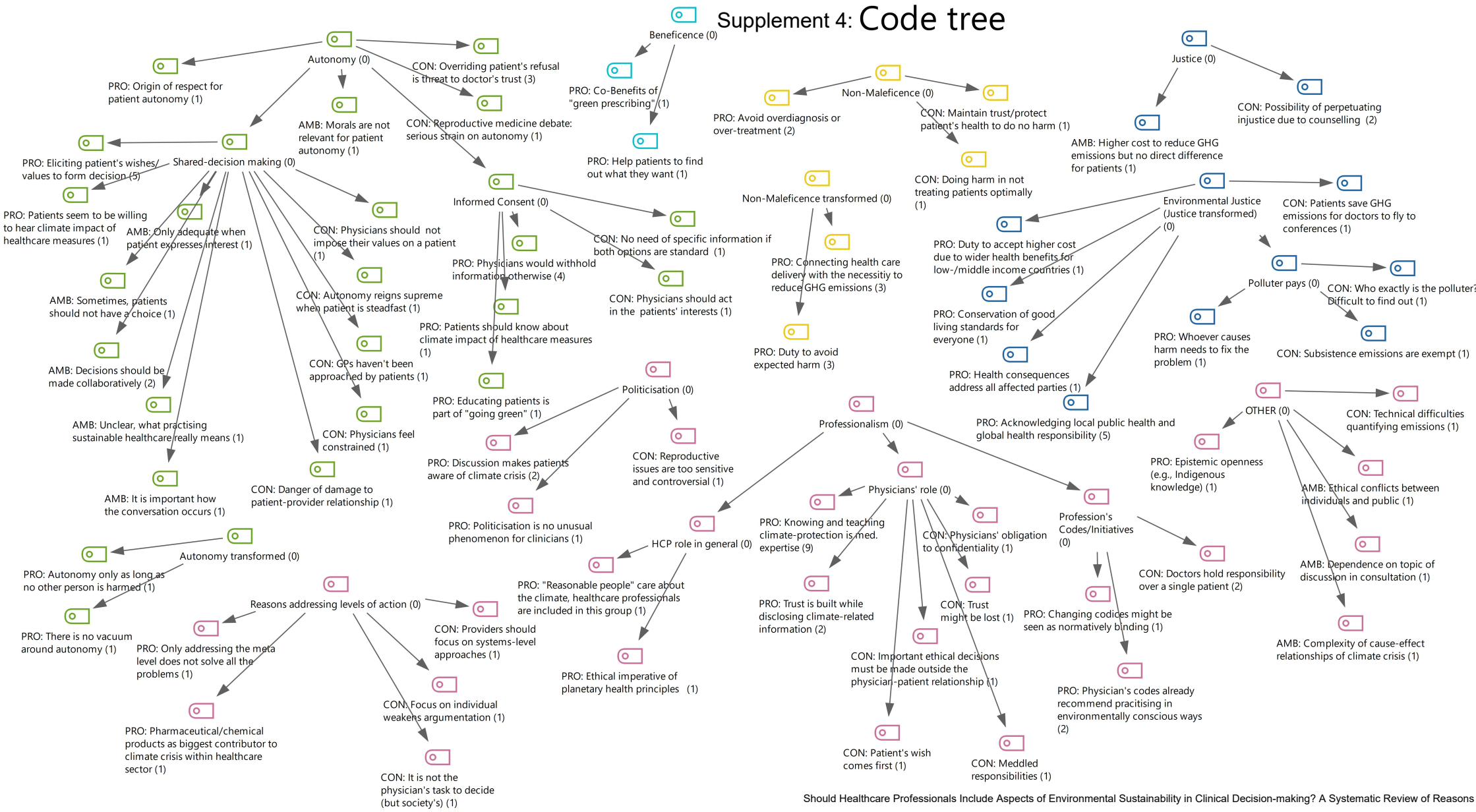

Supplement: Supplementary file 4 — Supplementary Material 4 [file 12910_2025_1230_MOESM5_ESM.pdf]
