# Supplementary material for: Should healthcare professionals include aspects of environmental sustainability in clinical decision-making? A systematic review of reasons
Source: BMC Med Ethics. 2025 Jul 3;26:78. doi: 10.1186/s12910-025-01230-4 (PMC12226885; doi:10.1186/s12910-025-01230-4)
Supplement: Supplementary file 5 — Supplementary Material 5 [file 12910_2025_1230_MOESM6_ESM.pdf]

Supplement 5: Reserve Checklist for: Should Healthcare Professionals Include Aspects of Environmental Sustainability in Clinical Decision-making? A Systematic Review of Reasons

	Title	Location where item is reported
1	**Title**: Identify the report as a systematic review.	Title
**Abstract**		
2	**Structured summary**: Provide a structured summary including, as applicable: objectives; ethics literature eligibility criteria; information sources; ethics literature appraisal and synthesis methods; publications included; synthesis of results; limitations of evidence; interpretation (conclusions and implications of key findings); funding; and a systematic review registration number.	Due to word count restriction only partially found in the summary
**Introduction**		
3	**Rationale**: Describe the rationale for the review in the context of existing knowledge.	Background
4	**Objectives:** Provide an explicit statement of the objective(s) or question(s) the review addresses.	Background
**Methods**		
5	**Eligibility criteria**: Specify the inclusion and exclusion criteria for the review (e.g. years considered, language, type of publication) and give a rationale.	Methods
6	**Search strategy:** Specify all databases, registers, websites, organisations, reference lists and other sources searched or consulted to identify publications. Specify the date when each source was last searched or consulted. Provide the rationale for using the information sources and, if applicable, present the full search strategy, including any limits and filters used, such that it could be repeated.	Methods
7	**Selection process**: Specify the methods used to decide whether a publication has met the inclusion criteria of the review, including how many reviewers screened each record and each publication retrieved, whether they worked independently, how disagreements were resolved and, if applicable, details of automation tools used in the process.	Methods
8	**Data extraction**: Indicate which sections of the publication were analysed and how the data were extracted from the publication. If applicable, state the software and details of automation tools used in the process.	Methods
9	**Identification of codes and themes:** Explain the process of assigning the codes, themes or items (e.g. inductive, deductive, a combination of deductive and inductive strategies) if applicable. If so, describe the process for the coding of data (e.g. line by line coding to search for concepts), including how many reviewers analysed each publication. List and define all other variables for which information was sought (e.g. participant and intervention characteristics, funding sources). Describe any assumptions made about any missing or unclear information.	Methods
10	**Quality appraisal:** Indicate whether a quality appraisal was performed and why, and if yes, outline the quality appraisal process and its results (e.g. how many reviewers assessed each study, did they work independently).	Methods
11	**Synthesis methodology:** Identify the synthesis methodology or theoretical framework which underpins the synthesis, and describe the rationale for the choice of methodology (e.g. thematic analysis, content analysis, critical interpretive synthesis, grounded theory synthesis, narrative synthesis). Describe any methods used to tabulate or visually display results of individual studies and syntheses.	Methods
**Results**		
12	**Publication selection process**: Describe the results of the search and selection process, from the number of publications identified in the search to the number of studies included in the review, with a flow diagram (including reasons for exclusions at each stage).	Results
13	**Characteristics of publications**: Present characteristics for which data were extracted and provide the citations for each publication included in the review.	Results
14	**Results of syntheses:** Present the results (e.g. new systematisation of issues or arguments) and reference publications as evidence.	Results
15	**Quotations**: Provide the original wording to illustrate themes, if applicable.	Results
**Discussion**		
16	**Summary**: Summarise the main findings and provide a general interpretation of the results in the context of other evidence; consider their relevance to key groups (e.g. health care workers, academics and other decision makers).	Discussion
17	**Strength and limitations**: Discuss the strengths and limitations of the publications included in the review and the review process itself.	Discussion
18	**Conclusions**: Discuss implications of the results for practice, policy and/or future research.	Discussion
**Other Information**		
19	**Registration and protocol**: Indicate if a review protocol exists, if and where it can be accessed (e.g. Web address), and, if available, provide the registration information including the registration number.	Methods
20	**Support:** Describe sources of funding for the systematic review and other support (e.g. supply of data); the role of funders for the systematic review.	Methods
21	**Competing interests:** Declare any competing interests of review the authors of the review.	End of discussion section
22	**Availability of data, code and other materials.** Report which of the following are publicly available and where they can be found: template information collection forms; information extracted from studies included; information used for all analyses; analytic code; and any other materials used in the review.	End of discussion section
